# Significance of myocardial fibrosis on cardiac function and morphology in severe aortic stenosis

**DOI:** 10.1186/1532-429X-15-S1-E109

**Published:** 2013-01-30

**Authors:** Sung Min Ko

**Affiliations:** 1 Radiology, Konkuk University Hospital, Seoul, Republic of Korea

## Background

Myocardial fibrosis occurs in aortic stenosis and is associated with long-term clinical outcome. Myocardial fibrosis is detected by delayed contrast-enhanced cardiac magnetic resonance. The aim of this study was to investigate the influence of myocardial fibrosis on left ventricular (LV) global function and morphology in patients with symptomatic severe aortic stenosis (AS).

## Methods

Eighty-nine patients (53 male, mean age 60 years) with pure severe AS (n = 36) or severe AS with mild aortic regurgitation (n = 53) were included in the study. All patients underwent transthoracic echocardiography (TTE), cardiac computed tomography (CCT) and cardiac magnetic resonance (CMR) and subsequent valvuloplasty operation. Patients were analyzed according to the presence and absence of myocardial fibrosis (midwall late enhancement) on CMR. The differences of cardiac function and morphology between two groups are statistically analyzed.

## Results

Myocardial fibrosis was observed in 37 patients. There were no differences in valvular morphology and clinical characteristics between two groups. Patients with myocardial fibrosis had higher aortic valve calcium volume score (2960 ± 1934 mm^3^ vs 1631 ± 1106 mm^3^, p = 0.0001) and calcium grade by CCT (p =0.0002), more severe AS [aortic valve area by CMR (0.72 ± 0.14 cm^2^ vs 0.83 ± 0.14 cm^2^, p = 0.0003), peak velocity (5.0 ± 0.7 m/s vs 4.5 ± 0.7 m/s, p = .002) and mean pressure gradient by TTE (59.5 ± 19.3 mmHg vs 49.4 ± 16.9 mmHg, p < 0.005)], higher indexed LV mass by CMR (90.0 ± 35.5g/m^2^ vs 63.2 ± 18.6 g/m^2^, p < 0.0001), lower LV ejection fraction by CMR (63.7 ± 14.0 vs 69.1 ± 11.6, p = .047), and larger indexed LV end-diastolic volume by CMR (93.7 ± 20.2 ml/m^2^ vs 79.7 ± 19.0 ml/m^2^, p <0.0001) compared with patients without myocardial fibrosis.

## Conclusions

Myocardial fibrosis is associated with more severe calcific AS, worse LV functional parameters and higher LV mass index in patients with severe AS. From these results, contrast-enhanced CMR may provide new strategies for the earlier diagnosis of severe AS with myocardial fibrosis.

## Funding

None.

